# Understanding resilience among migrant women in a humanitarian reception center: a qualitative study

**DOI:** 10.3389/fpubh.2024.1372900

**Published:** 2024-09-30

**Authors:** María del Mar Jiménez-Lasserrotte, María Angustias Sánchez-Ojeda, Gloria Vázquez-González, María Dolores Ruiz-Fernández, Azahara Peña-Rodríguez, Isabel María Fernández-Medina, José Granero-Molina

**Affiliations:** ^1^Department of Nursing, Physiotherapy and Medicine, Faculty of Health Sciences, University of Almeria, Almeria, Spain; ^2^Department of Nursing, Faculty of Health of Sciences of Melilla, University of Granada, Melilla, Spain; ^3^Department of Nursing, Faculty of Nursing, Physiotherapy and Podiatry, Universty of Sevilla, Sevilla, Spain; ^4^Spanish Red Cross, Almería Provincial Office, Almería, Spain; ^5^Facultad de Ciencias de la Salud, Universidad Autónoma de Chile, Temuco, Chile

**Keywords:** irregular migrant women, psychosocial health, qualitative study, resilience, social support

## Abstract

**Background:**

The European Union receives thousands of irregular migrants and refugees annually. Irregular migrant women are admitted to Humanitarian Reception Centers. These migrants face multiple adversities on their migration journey, and resilience is key to coping with process. The aim of this study was to describe and understand irregular migrant women’s experience of resilience when living in humanitarian reception centers.

**Methods:**

Descriptive qualitative study. In-depth interviews and a focus group were carried out with 21 migrant women from different African countries, with an average age of 31.8 years. Thematic analysis was used to analyze the qualitative data using ATLAS.ti computer software.

**Results:**

Three main themes emerged: (1) Irregular migrant women in transit: extreme vulnerability. (2) Migration support networks. (3) Promoting irregular migrant women’s resilience.

**Conclusion:**

The harshness of the migration process tests the resilience of irregular migrant women, who are a vulnerable group at high risk of social exclusion. Their time in humanitarian reception centers is significant in their process of developing resilient behaviors. The multidisciplinary resources of the humanitarian reception center help the irregular migrant women in their personal development.

## 1 Introduction

According to the International Organization for Migration, more than 280 million people were involved in global migratory movements in 2020, many of which were irregular (1). Irregular migration refers to the cross-border movement of persons or groups not legally authorized to enter or stay in the country of destination (2). The European Union (EU) receives thousands of irregular migrants (IMs) and refugees annually, who are fleeing war (3), political conflict, religious conflict (4) or sexual violence, while others are in search of better living conditions (5). This type of migration poses a humanitarian, epidemiological and public health challenge in destination countries (6). Like Italy, Greece or Turkey (7), Spain is a Mediterranean country that receives IMs (8). After emergency care (9), IMs are admitted to humanitarian reception centers (HRC) until their fate is decided (10). IMs face multiple adversities on their migration journey to the EU, and resilience is key to coping with the process. Little is known about IMs’ own experience of resilience, which is influenced by individual, social and cultural factors (6).

Irregular immigration to the EU is progressively increasing; 41,861 IMs (40,326 by sea) arrived in Spain in 2020, 5–9% of whom were irregular migrant women (IMW) (11). It is becoming increasingly common for IMW to migrate with minors and/or while pregnant (7) and they are a highly vulnerable group (12). Land movements and sea crossings are extremely dangerous (12, 13) and IMs risk their lives on the journey (3). The harshness of the migratory process has a negative impact on the health of IMs, especially on IMW and children, whose physical, psychological and social well-being is affected (14). In addition to being faced with a lack of hygiene, food or health care, IMW and children are at greater risk of violence, sexual exploitation and human trafficking during the migration journey (15, 16).

After crossing the Mediterranean Sea and disembarking in Spain, IMs receive emergency care from specialized teams (9); particular attention is given to vulnerable groups, such as IMW and children (17). After receiving emergency care, IMs remain in police custody for a maximum of 72 h to carry out administrative procedures and determine their extradition. IMW and children are placed in HRCs for a maximum of 3 months. These centers are managed by NGOs and located all over the country. Their main objective is to house and cover the basic needs of the IMs, who do not have the resources for a dignified life in the host country (10). As well as providing legal counseling, the HRCs establish care plans for IMW and children to cover their basic, psychological and social needs. The HRCs actively work to encourage the IMW’s autonomy, facilitate their integration into the host society and improve their resilience (18).

Resilience is the human capacity to cope with life’s adversities. It involves establishing protection measures at both an individual and community level (19), as well as using coping and adaptation skills to overcome a challenge (13). It is the ability to deal with difficult personal or environmental situations while maintaining positive outcomes (20). The capacity for resilience develops throughout life or in response to a critical life situation, the latter being the case for IMW. Irregular migration has a significant impact on the IMW’s physical and psychological health, thus affecting their resilience (21–23). The experience of being a victim of violence or trafficking, or adapting to a new environment (20), puts IMW’s resilience to the test (24). Cultural values, spirituality and empowerment can be key factors in facilitating IMW’s integration into the host society (25). Resilience factors have been studied in socially marginalized African populations (21). IMW’s resilience in host countries is associated with their socio-cultural and family background (26), faith, values, level of empowerment and personal skills (25). Although some studies claim that being a woman and a foreigner favors resilience (27), it is a challenge for IMW to raise children shortly after arriving in a new country (15, 28). Little is known about the factors and resources involved in the IMW’s process of building resilience during their time in HRCs. By understanding which factors influence the resilience process of IMW, we can respond to their needs better. Our research question was: how do IMW experience resilience during their time in the HRC? The aim of our study was to describe and understand IMW’s experience of resilience when living in HRCs.

## 2 Materials and methods

### 2.1 Design

A descriptive qualitative research design has been used. This approach allows for an in-depth description of poorly understood phenomena, such as resilience in IMW, by exploring the participants’ own experiences (29). The researchers place themselves in a constructivist paradigm in search of a shared understanding of the phenomenon, by interpreting the results as a fusion between the accounts of the participants and the experience of the researchers. According to Colorafi and Evans (30), this design is suitable for investigating how people feel and act in particular health settings. Consolidated criteria for reporting qualitative research (COREQ) have been used (31).

### 2.2 Participants and context

The study was carried out in an HRC in southern Spain. The participants were recruited by HRC psychologists through purposive sampling. Inclusion criteria were: IMW over 18 years of age and minimum stay of 3 months in an HRC in Spain. Exclusion criteria: refusal to participate in the study, not speaking a language known to the researchers (Spanish, English, French, Arabic). Twenty three IMW were invited to participate and 2 refused because they were not psychologically ready. The final sample consisted of 21 IMW with an average age of 31.8 years. The socio-demographic characteristics of the participants can be found in Table 1.

**Table 1:** The socio-demographic characteristics of the participants (*N* = 21).

Participant	Age	Country	Children	Pregnant	Religion	Months in HRC	Family unit	Level of education
IDI-1	46	Morocco	4	No	Muslim	22 months	Woman, man, 1 son	No studies
IDI-2	31	Morocco	1	No	Muslim	5 months	Woman, 1 son	No studies
IDI-3	38	Morocco	1	No	Muslim	5 months	Woman, 1 daughter	No studies
IDI-4	36	Morocco	4	No	Muslim	4 months	Woman, man, 4 sons	No studies
IDI-5	47	Morocco	1	No	Muslim	17 months (2nd time)	Woman, man, 1 daughter	No studies
IDI-6	28	Algeria	2	No	Muslim	5 months	Woman, 1 son	Secondary
IDI-7	25	Algeria	3	No	Muslim	7 months	Woman, 3 sons	Secondary
IDI-8	40	Ivory Coast	2	No	Christian	12 months (2nd time)	Woman, man, 1 daughter	No studies
IDI-9	25	Senegal	2	No	Muslim	7 months	Woman, 1 daughter	Primary
IDI-10	35	Morocco	2	7 months	Muslim	3 months	Woman, man, 2 daughters	No studies
IDI-11	21	Nigeria	1	No	Christian	9 months (2nd time)	Woman, man, 1 daughter	Primary
IDI-12	24	Algeria	1	No	Muslim	4 months	Woman, 1 daughter	Primary
IDI-13	48	Morocco	2	No	Muslim	5 months	Woman, 1 son	Primary
IDI-14	23	Algeria	1	No	Muslim	6 months	Woman, man, 1 daughter	Secondary
IDI-15	32	Senegal	6	No	Muslim	4 months	Woman, 1 daughter	No studies
FG-1	24	Algeria	2	No	Muslim	7 months	Woman, 2 sons	Secondary
FG-2	25	Morocco	2	No	Muslim	3 months	Woman, man, 2 sons	Primary
FG-3	23	Algeria	1	No	Muslim	3 months	Woman, 1 son	Primary
FG-4	40	Algeria	3	No	Muslim	10 months	Woman, man, 1 daughter	Primary
FG-5	24	Algeria	1	No	Muslim	6 months	Woman, man, 1 daughter	Secondary
FG-6	34	Morocco	2	No	Muslim	7 months	Woman, man	Primary

FG, Focal group; IDI, In-depth interview.

### 2.3 Data collection

A focus group (FG) with 6 IMW from North African countries and 15 in-depth interviews (IDI) with IMW from different African countries were conducted between April and June 2022. The FG lasted 79 min. It was followed by 15 IDIs with an average duration of 53 min. The aim of the IDIs was to explore the issues in further detail. All sessions took place in HRC meeting rooms and were carried out by trained researchers and cultural mediators who spoke the participants’ languages and used a script of questions (Table 2). The principal investigator of this study has extensive experience in emergency care for IMs. She has a pre-understanding of the resilience process of IMWs from observing them in other phases of the care process and exchanging information with her peers in HRCs. The collaboration between the researchers and HRC workers was positive; they identified the most appropriate times for data collection and facilitated an empathetic relationship with the participants. An interview script was designed to encourage participants to share their experiences. It was developed based on previous studies related to the topic and was validated by professionals from the HRC, who are experts in caring for migrant women. The interview protocol was tested prior to conducting the interviews so that they would feel like a conversation. The data and interpretations of the researchers were given back to the participants to obtain feedback on the research results. Before starting data collection, the protocol was explained, data confidentiality was assured, and informed consent was signed. Data collection ended when no new information was provided when analyzing the data, as data saturation had been reached (32).

**Table 2:** Interview and focus group script.

Phase	Themes	Content/Possible questions
Introduction	Objective	To understand the resilience of African women who arrive in Spain across the southern border and live in a humanitarian reception center.
	Ethical considerations	Inform about voluntary participation, recording, consent, the possibility to withdraw, and confidentiality.
Beginning	Initial question	Please could you tell me about your experience of migrating to Spain?
Development	Interview and focus group script	You then went to live at a migrant reception center. Tell me about this place you went to live. How do you feel living there? What do you think helped you to advance in the migration process despite all of the difficulties you have faced? Which of these factors do you consider to come from within you and which are external? Have you had people or resources to help you recover? What motivates you to move forward?
Closing	Final question	Would you like to add anything else about what we have spoken about?
	Thanks	Thank them for their time and tell them that they can contact us if needed.

### 2.4 Data analysis

The FG and ID recordings were transcribed and analyzed following the phases of thematic analysis described by Braun and Clarke (33): (1) Familiarization with the data: data were analyzed as they were collected, for an early understanding of the experiences; (2) Generation of initial codes, prior to grouping into categories; (3) Inductive analysis to create possible themes; (4) Review of themes: two researchers independently verified transcripts and results, sub-themes and themes; (5) Defining and naming themes: analyzing and refining the details of each theme. (6) Report writing: selecting examples of themes and sub-themes, relating analysis to the research question and generating a final report. Data analysis was carried out using Atlas Ti.9.3 software.

### 2.5 Rigor

To ensure rigor, Guba and Lincoln (34) criteria were adopted. Credibility: for data triangulation, the researchers performed the analysis individually and then reached a consensus. The researchers are social and healthcare professionals with extensive research experience in IMs. Transferability: detailed information on participant characteristics, lived experiences and context was provided. To ensure reliability and confirmability, detailed descriptions of each step of the research process were provided, along with an audit trail of transcripts, categories and coding memos. An external researcher with more than 5 years of experience in IMW care reviewed the findings. The authors kept a reflective journal about how their values and preconceptions might affect the research at each stage. Quotes from the transcripts of the participants’ experiences were incorporated into the findings.

### 2.6 Ethical considerations

Approval was obtained from the Ethics and Research Committee of the Spanish Red Cross (No.: CR-20-01). The research was conducted in accordance with the ethical principles of the Declaration of Helsinki. Participants were asked for written informed consent and permission to record the interviews. All records respected current legislation on personal data protection.

## 3 Results

All participants were IMW from African countries (*N* = 21). Their mean age was 31.8 years (SD = 8.7). The mean length of time they spent at an HRC was 7.2 months. Three themes and eight sub-themes emerged from inductive data analysis, providing insights into the experiences of the IMW’s resilience at the HRC (Table 3).

**Table 3:** Themes, subthemes, and units of meaning.

Theme	Subtheme	Units of meaning
Irregular migrant women in transit: extreme vulnerability	Resilience starts in the country of origin	Unemployment, poverty, violence, persecution, lack of rights, difficult childhood, patriarchy.
	A dangerous journey that puts resilience to the test	Persecuted, settlements, police control, fear, journey on a small boat, near death experience, giving birth on board, land crossing.
Migration support networks	Rescue and arrival: a storm of emotions	Rescue priority, social and health care, police custody, fulfilled objective, happiness, rebirth, fragility, dependence.
	The reception center: my new family	New home, safety, happiness, communal living, family, needs met, freedom, humanity.
	Resources for irregular migrant women’s development and autonomy	Education, love, personal development, work permit, language, self-care, cultural melting pot, legal standing, social inclusion, family, intervention group.
Promoting irregular migrant women’s resilience	Introspection skills: seeking inner strength	Empowerment, future, overcoming, autonomy, optimism, bravery, desire to live, composure, self-esteem, solidarity.
	Growing out of adversity: spirituality and social/job support	Strength, leadership, brave woman, freedom, spirituality, independence, entrepreneurship.
	Family and care network: the foundation for resilience	Improve children’s and family’s life, family reunification, future, family support.

### 3.1 Irregular migrant women in transit: extreme vulnerability

Immigration is a phenomenon that involves multiple countries. Although the majority of IMs are still men, the feminization of poverty, commercialism and patriarchal domination are factors that have contributed to the increase of IMW. Women in precarious situations in their countries of origin take a bold step to take charge of their lives and initiate the migration process.

#### 3.1.1 Resilience starts in the country of origin

The participants had a difficult life in their countries of origin. Poverty, violence and vulnerability pushed the IMW to embark on the migration journey. The women were aware of the dangers of the journey, so it was a conscious yet difficult decision. The process of resilience started from the very first moment when IMW drew on all of their personal resources to overcome the long migration journey. The IMW were pushed to leave their homes due to job insecurity, domestic violence or political problems, most of which had their partners to blame. As one participant noted:

“*My husband wrote a lot on the internet, he wrote on forums … He attacked the king, the authorities … and that reached a lot of people. Then the police came to the house to look for him”* (IDI*-*Ivory Coast).

The IMW had been in a vulnerable position within their families and had long endured family and marital pressures, including personal and sexual harassment. These situations related to their personal, home and social environment played a defining role in their resilience. Seeking safety helped the IMW to grow through adversity and it shaped their future decision-making process.

“*The reason I decided to leave my country was mainly because I had been married before. I decided to get divorced because the situation wasn’t good, and then I was harassed by my ex-husband.”* (IDI*-*Morrocco).

The participants described the need to leave their countries of origin due to threats or childhood abuse. They experienced isolation, vulnerability and social exclusion; they were afraid for themselves and their children. The IMW were aware of the adversity they had experienced during their childhood and wanted to ensure that their children would have a safe future. This vulnerability influenced the decision they made, shaped their personalities and developed their resilience.

“*My family was fine, but … I had issues with my father, which nobody knew about until I came to Spain. I told my mother and she asked me why I had not told her before. I went through a lot of… but he was my father, and I ran away from home…”* (IDI-Nigeria)

#### 3.1.2 A dangerous journey that puts resilience to the test

The journey from their countries of origin to the Moroccan or Algerian coasts involved overcoming multiple obstacles. The IMW and their children suffered from hunger, exhaustion and violence. Some fell victim to criminal organizations looking for financial gain. They were also used as cheap labor or undercover prostitutes in settlements in North African countries, where they were waiting to cross the Mediterranean. As one participant said, these hardships were compounded by traveling with children, or being pregnant during the journey.

“*To leave Algeria you have to pay. The journey is tough, I was pregnant, and the organisers do not want small children. There were many boats leaving and I had tried to make the trip since September, but I could not because of the children. I had to wait until December because they do not give you your money back”* (IDI-Algeria).

Police authorities exercised tight control of border areas, so the IMs used isolated areas as hiding places. The participants explained how they hid in settlements when they reached the coast to avoid the Moroccan police. The IMW highlighted the harsh reality of the time they spent waiting for the conditions to be favorable enough for them to cross the sea. Resilience is a dynamic process through which IMW acquire resilient qualities, skills to understand the meaning of adverse situations and useful lessons for the future.

“*The conditions where we were waiting were very bad, we had nothing to eat… They hid us in a hole under the mountains. We were there for a week until they told us to get on a Zodiac [inflatable boat]. There were so many of us, but you could not back out! My son had a fit, he was very thirsty and could not breathe!* (IDI-Ivory Coast).

IMs do not cross the Mediterranean Sea together; the migration journey may involve their family being split up, resulting in each member making the journey separately. This increases the IMW’s exposure to risk and makes them more concerned about the fate of their family members. Nevertheless, IMW often stay with their children; this lonely and isolating situation leads to an innate motivational strength and resilience to overcome the hardship of the waiting period. The expectation of being reunited with their families gave the participants the strength to overcome the extreme conditions of making the journey on a small boat.

“*My husband was lucky enough to cross before me, I was left alone with my son. We were at sea for five days and I thought I was dying, I felt awful, I was wet, vomiting. Now I see the sea and it scares me, imagine being there at night when you cannot see anything.”* (IDI*-*Morrocco).

During the perilous journey, the IMW were scared and at risk. If they were pregnant and/or accompanied by children, the situation was even more difficult. One participant remarked how she gave birth to her daughter at sea. At the time of departure, she started having uterine contractions yet decided to go ahead with the journey. Another woman with midwifery skills traveled with her to assist with the birth, in exchange for her and her child traveling for free. This is how one IMW described the situation:

“*When I gave birth, I was very scared… I thought I would not survive. It was very cold, the water from the waves was hitting us, we were wet, very cold! On the way there I thought the baby had died. I looked at her, wet, I thought she would not make it to Spain alive!”* (IDI-Algeria).

### 3.2 Migration support networks

For the IMW, reaching land or being rescued at sea means fulfilling their migration dream. When IMW disembark in Spain, they face strict police controls and remain in custody until they obtain a file for processing deportation. This administrative procedure determines their future, whether it is to start a new life in Spain or return to their country of origin. Various restrictions negatively affected the physical, psychological and emotional state of the IMW, who felt once again controlled and deprived of their freedom. In this phase, social and health support networks are key as they help the IMW with the adaptation process and administrative procedures.

#### 3.2.1 Rescue and arrival: a storm of emotions

IMW and children are the first to be rescued, and despite their awful experiences, the inner strength of the IMW is remarkable. Most of the participants described feelings of happiness, optimism, safety and hope. They felt joy at fulfilling their dream of starting a new life. Their spirituality helped them to trust in God, which made them feel confident and reassured.

“*It was a very tough experience; the boat was small and the engine was very weak. When we saw the rescue boat [Salvamento Marítimo], I could not shout, but I cried with joy. They rescued us at sea, first my daughter and me (the only women). Thanks to God we arrived, and the sadness went away… I am no longer sad, I am a happy woman”* (IDI*-*Morrocco).

Health and social care upon arrival complies with protocols for rescue, humanitarian care, health and police custody. When the IMW first arrived, they went to humanitarian care units for an initial biopsychosocial health assessment. Information was obtained about their family and migration plan, and a referral to an HRC was arranged. As one woman noted, despite feeling exhausted, happiness and hope prevailed. This reinforced her will power and personal strength to overcome the harsh situations she had experienced.

“*We had arrived in another country, we were hungry, thirsty, cold…, but it did not matter, we were excited and hopeful”* (IDI-Senegal).

However, when they were subjected to police procedures, they felt fragile once again. When they were taken to the police station for identification and return/assignment to an HRC, their previous feelings of vulnerability were reawakened.

“*When we were handed over to the police, we did not know what was going to happen to us, if they were going to send us back to our country or keep us in Spain… We were afraid that they would send us back to Morocco, that we would have suffered for nothing.…”* (IDI-Algeria)

#### 3.2.2 The reception center: my new family

After the police custody phase, IMW can go to an HRC run by NGOs. This facility aims to cover their basic needs, provide accommodation and promote social integration. The IMW admitted to an HRC felt safe, happy and resilient. They described how they felt free and recognized as people; they started to regain the identity they had lost during migration.

“*The feeling is happiness, to be able to rest, sleep well…in peace. They have always treated me well, you feel like they are all looking out for you, they look at you with kindness and affection”* (IDI*-*Morrocco).

Aware of the difficulties of integrating into the host country, the IMW acknowledged the NGOs’ work and thanked them for their efforts. The participants considered their treatment in the HRC as a fundamental aspect of humanitarian aid. These facilities became their new home and the workers their new family. Furthermore, living with others met their needs for social interaction.

“*If we had not had that social support … we would not know anything, we might be on the street. When we arrived at the centre, we were warmly welcomed by everyone. They teach us to defend ourselves for when we leave. We are very happy, they love my daughter… for us it was like a family”* (FG-Algeria).

#### 3.2.3 Resources for irregular migrant women’s development and autonomy

The participants considered the main support resources to be the Spanish classes and job search service provided. They also highlighted the schooling process for their children, which is essential for their education and integration into the host society. One IMW described how the support received fosters a sense of belonging and connection:

“*They help us to do our CVs, to learn the language, progressively get to know people and find work. The most important thing for me has been my children’s schooling … seeing them at school makes me happy, they learn Spanish very quickly!* (IDI-Algeria).

As well as social care, HRCs offer administrative and legal support in the identification phase of IMW arriving on the coast. This procedure is necessary to regularize their status. The process of obtaining a residence and work permit is slow, and IMW are afraid of having it denied. This is how one participant expressed her gratitude for the support she received to regularize her administrative situation:

“*Now my goal is to get papers. In the centre they are helping me, they accompany me to the police station, to the immigration office, … I thank them for not leaving me alone. Thanks to them everything was easier!”* (IDI-Senegal).

Living at an HRC with people from other countries and cultures was a very enriching experience for the participants. This melting pot of nationalities taught them to live together, to relate to one another, and share their experiences. It allowed them to establish personal connections and feel that they were part of a family. As one participant said, the HRC promoted their sense of belonging:

“*There were a lot of very different people, from different countries, speaking different languages, it was a good experience. I met a new family, the family of the centre where I live.”* (IDI*-*Morrocco).

The IMW highlighted their gratitude to the HRC team for making them feel loved and for supporting them in their personal development. They also contributed to the women being able to leave their difficult past behind them and find happiness. They arrived at a warm and welcoming place where they could recover and achieve their goals.

“*From the beginning they took care of us, they spoiled us…. They treated me well and I felt like part of a family. On the last day of Ramadan, they gave me presents, … I even cried. They gave my children presents, … nobody in Algeria does that. They also tried their best to make us learn at our own pace, we had everything”* (IDI*-*Algeria)

### 3.3 Promoting irregular migrant women’s resilience

This theme describes the IMW’s personal resources to cope with adverse events in their lives. The IMW have exhibited resilient behaviors that demonstrate inner strength to fight against a lack of basic rights, sexism, violence, unemployment or administrative irregularity. The participants developed an attitude of perseverance and were unwavering in their support for their families and themselves. They overcame their fragility by finding strength and resilience.

#### 3.3.1 Introspection skills: seeking inner strength

The IMW highlighted self-confidence and self-esteem as essential internal forces for moving forward. They had been autonomous from a young age as they were forced to work to help the family. In the HRC they learned to be more independent and stand up for themselves. They participated in workshops that promoted the process of transforming power relations and combating inequality. This is how one IMW described how she was able to find work and establish a stable, dignified and safe life in Spain:

“*Support and self-confidence are fundamental to overcome this, … it’s not the same, a divorced mother in Algeria encounters social rejection. I have studied, I have self-confidence, I have fought not to go down the wrong path. I am not the only single mother who is like this! I always thought this, I encourage women to fight, not to depend on anyone, to fight for what is theirs!”* (IDI-Algeria).

The participants’ accounts revealed that courage and composure are attributes that help them to cope with adverse situations. The IMW stated that they had to have patience and willpower, as well as follow certain rules if they wanted to reach their goal. Knowing that gender differences make the process more difficult, they have to be persistent and not get frustrated at every obstacle.

*“For men it is easier; travelling, working and making money, … for women it is not easy. In Nigeria they want money fast, they get into dirty business and trouble because they are not patient.”* (IDI-Nigeria).

A patriarchal society defines the psychological characteristics of both men and women, as well as the social roles they need to fulfill. The IMW were victims of violence in their countries of origin, where there is no social protection system, and where they were invisible and victimized. The IMW became more aware of themselves in the HRC; they learned to value and love themselves after seeing and feeling that their rights were respected.

“*I have seen the difference in the treatment of women in Algeria and Spain. I see the rights of women and I like it, … also how they support children.”* (IDI-Algeria).

The IMW recounted very difficult experiences, such as being separated from loved ones, struggling to survive, or receiving threats. They worked through these issues by finding the courage and will to overcome adversity, which was supported by the multidisciplinary team. The IMW thanked them for promoting a sense of social belonging, and for accompanying them and empowering them in their new life project.

“*I’ve suffered a lot, I’ve had a tough past, I’ve failed at times and … I’m moving on. I said to myself, I cannot live here, this is not my last stop, I have to move on and live where I can be happy. You have managed to give me hope, to know that I have a future,… it’s very positive”* (IDI-Ivory Coast).

The desire to help others was another motivation to improve, work hard and achieve their goal. Engaging with empathy and helping others were positive coping mechanisms in the face of adversity. This also contributed to increasing their resilience.

“*When I have a job, I will use it to help friends, family, people. I want to help people who do not have food or a place to live, … like they have helped me.”* (IDI-Senegal)

#### 3.3.2 Growing out of adversity: spirituality and social/job support

The experience of living through difficult situations had an impact on the IMW’s process of resilience and allowed them to grow on a personal level as a result. Facing hardship and finding the way to move forward has taught them how to adapt to different situations according to their own goals. The support of the HRC team has also helped them to become stronger:

“*You have made me strong; because of you and your advice I have been able to achieve even more. Now I have hope, that I had lost at some point. Now I am going to fight, to live, to do everything I can to integrate myself and help my family.”* (IDI-Senegal).

These courageous and resilient women use their strength and reluctance to suffer to heal emotionally and move forward. Finding a job was another of the fundamental factors for gaining personal autonomy. The IMW have an entrepreneurial spirit and have not come this far to give up. They would rather find work and get ahead, a process that was facilitated by the HRC.

*My dream is to have my own house, my own space, to educate my daughter. To be a strong and in-dependent woman…to have a company with our own account. I want workers and to help their families!”* (IDI-Nigeria).

Their spirituality and desire to improve the lives of their loved ones were factors that reinforced the resilient nature of the IMW. Their religious beliefs and trust in God gave meaning to their lived experiences and suffering. For many of the IMW, their faith enabled them to migrate.

“*God has helped me, to have faith, courage, to pray. When I left, I told the ones I left behind that everything was possible if you prayed.”* (IDI-Nigeria).

#### 3.3.3 Family and care network: the foundation for resilience

The social support network provided the IMW with strength to recover from adversity and protect their families, thus giving meaning to their experiences. The participants were able to resist, learn and grow. They found the way to transform pain and fear into new opportunities, which represented a turning point in the endeavor to start a new life. They were happy to have support and a roof over their heads, but most of all for their children to grow up in a free country.

“*For me, the most important thing are my children, the biggest change is that my children are here. Growing up here is not like there (Algeria). They are free, they can grow up in a context of equality…that*’*s really important”* (IDI-Algeria).

Inequality, sexism, abuse and the possibility of improving their lives and those of their families motivated the participants to embark on the migration journey and overcome any adverse situation. Their love for their children was the strongest driving force behind their struggle.

“*The main reason for travelling was to get my kids away from my ex-husband and his family. If I had stayed there, we would not be together”* (IDI*-*Morrocco).

The motivation to create a better future provided them with the determination to fight and overcome the difficulties they faced. The IMW know how important the support from their families, friends or NGOs was to achieve this as it gave them the necessary confidence.

“*When I go through hard times, it helps to know that I have people who care about me. Wherever I am, my Mum will be praying for me. Now I can succeed in a life with friends and family, a happy life”* (IDI-Nigeria).

## 4 Discussion

The aim of our study was to describe and understand IMW’s experience of resilience when living in an HRC. A qualitative descriptive study has allowed us to understand the experiences, resources and strengths of IMW to develop resilience and start a new life in the host country. IMW initiate the migration process for various reasons: financial motives (35), gender inequality (7), lack of safety or family-related problems in their countries of origin (36). They are also pushed to cross borders and risk their lives due to poor living conditions, a lack of social protection, fear for their children and a desire to give them a better future (6, 9). The process of resilience begins in the countries of origin, where IMW experience traumatic situations such as violence, ablation (35) and control by human traffickers (37). The participants in our study described how they were able to become aware of negative lived experiences, to acquire resilient qualities and to cope with extreme vulnerability (38). Although some studies show that exposure to adverse environments can lead to depression or anxiety (20), these IMW developed motivational strength to overcome adversity and improve their lives (39).

Although many people die in the attempt (8), making the journey on a small boat is the most common means used by IMs to reach Spain (9). According to Ponce-Blandón et al. (12), our study corroborates that IMW experience hardship and fear; they fall ill, get detained in settlements or even give birth at sea. IMW require support resources to adapt and recover from these adverse situations (38). According to the United Nations Human Rights Council (UNHRC) (40), it is important to ensure the protection of IMW who have suffered trauma and sexual violence. In this regard, the results of this study corroborate that institutions such as Salvamento Marítimo (Maritime Rescue), the Red Cross or the police provide rescue services and health care that fulfill the basic needs of the IMW (6, 41). The care provided to the IMW made them feel grateful, happy and optimistic for having fulfilled their dream of reaching the host country and ensuring a better future for themselves and their children (36). Concurring with Aubé et al. (15) and Villacieros (37), there are key driving forces for IMW to persist with their migration process and increase their resilience. These include protective factors, such as positive projection, willingness to give meaning to their lives, growing in the face of adversity and turning to their faith.

The participants described having experienced a range of feelings since their arrival in Spain and admission into the HRC. As in other studies, they allude to dreaming of happiness (35), being safe (42), and no longer feeling vulnerable (43). The difficulty of the IMW’s integration process during this first phase in the HRC raised concerns among the researchers. However, they were reassured by the high professionalism and expertise of the HRC staff in their support of IMW. The support networks available facilitate the integration of IMW, contributing to the resilience process (15). The bidirectional relationship between IMW and social institutions helps IMW to integrate, obtain residence permits and overcome inequality (44). Despite the challenges they face (45), IMW highly value the resources offered at the HRC, such as Spanish classes (27), legal services or help in their job search. After spending time at the HRC, they feel alive and are open to new experiences; they strive for a better life and the ability to settle down.

Our findings highlight optimism, spirituality, social support, support from family and entrepreneurship (36, 46) as key factors influencing resilience. Resilient people are optimistic about the world; they perceive and confront difficult situations as a challenge (47). According to Abraham et al. (38), the ability to handle stressful situations and understand their importance is paramount. The IMW were able to learn from their experiences of gender inequality, lack of freedom or lack of dignity, which they identified as reasons for losing hope. We concur with Aubé et al. (15) that the IMW’s time in the HRC reduced gender disparities, empowered them and boosted their resilience. Religion and spirituality helped the participants by providing them with a meaningful framework within which to overcome difficulties and increase their resilience. Their faith provided them with a sense of reassurance that allowed them to overcome adversity, thus acting as a shield of protection (46). The belief in overcoming challenges, finding work, reuniting their family or providing a better future for their children, helped the IMW in the face of adversity (48). The IMW shared their empowerment strategies, which included setting up small businesses to gain financial security and live a dignified life (21). According to other studies, HRCs promote an environment for cultural diversity and safety, as well as a space for the IMW to adapt their values and aspirations (36). It is important to identify cultural variables and specific socio-political contexts, because both factors play a crucial role in the development of resilience (49, 50). Studies indicate that societies that foster gender equality improve women’s well-being, problem-solving skills and resilience (51). Understanding these pre-existing factors of resilience provides insight into the ideology of IMW and the meaning they attach to adversity (50). It is not only about protecting IMW, but also about providing them with the tools to facilitate their integration into the host community (15). These include learning the language of the host country, finding a job (18) or getting their children into school (52). Resilience in IMW should be understood as a protective factor; it is the ability to use their negative experiences for their own benefit (45). The WHO health system framework states that countries should promote health system planning that includes sufficient resources to strengthen resilience (49). An important strength of this study is how it corroborates this need, which was articulated by the IMW themselves, who are the ones who have experienced the process first-hand.

The reflexivity of this study was based on making decisions in a reflective and collaborative manner (53). M.d.M.J.-L., the principal investigator of this study, has extensive experience in emergency care for IMs and has a pre-understanding of the IMW’s resilience process. She understands the process of resilience as a slow and complex adaptation, influenced by cultural immersion and the need to escape from the unseen threat of traffickers. The first step in her personal reflexivity involved managing assumptions about how her routine work with IMs might influence the results of the study. The researchers kept a reflective diary throughout the research process; at various stages during data collection and data analysis, they used memos and field notes to record their assumptions, preconceived ideas, changes in attitude or contextual interference. By reflecting on their findings and going back to the participants’ narratives, they were able to develop interpretations of the IMW’s experience of the resilience process. It is worth noting that IMW were afraid to answer certain questions, especially in the focus group, due to a lack of confidence in sharing their experiences.

### 4.1 Limitations

This study used purpose sampling and only included IMW from different countries arriving in Spain across the southern border. As all the study’s participants were in the same HRC, the findings may not be representative of the entire population of IMW arriving in Spain and Europe, thus limiting the transferability of the results to other contexts. Furthermore, the IMW struggled to answer our questions; the first few days they were still highly affected by the arduous migration journey, resulting in some interviews having to be postponed, which could have influenced the results. Moreover, the unstable situation of these women, still affected by the fear they experienced during their migration journey, could have limited their responses. Lastly, although the mediators spoke the same languages as the IMW, some local dialect words may not have been fully understood or transcribed well. Power dynamics can lead to communication barriers and the IMW may not have felt fully comfortable expressing themselves. Nevertheless, the participants’ involvement in this study, alongside the support of HRC researchers and staff, was linked to the progressive improvement of their wellbeing. In order to mitigate bias, future research could consider purposive sampling based on age group, educational level, length of time in the HRC or point of arrival in Spain.

## 5 Conclusion

Harsh living conditions in their countries of origin and traumatic experiences during the migration process put the IMW’s strength and resilience to the test. This process begins in their countries of origin and continues beyond being transferred to the HRC. The results of this study suggest that IMW have conflicting feelings: the happiness of having arrived in the host country and the uncertainty of whether or not they need to return to their country of origin. IMW are a vulnerable group at high risk of exclusion due to their health status and administrative situation. This is compounded by precarious living conditions, unequal access to services, language and cultural barriers, and their unfamiliarity with the system. Many IMW are referred to HRCs, a fundamental pillar of support that allows them to regain their self-esteem and confidence. They also facilitate their path to freedom and integration. The participants demonstrated resilient behaviors; their independence, optimistic attitude, courage and calmness have all been key to overcoming adversities. The IMW are motivated to strive for a better future for their family and to build a career. The multidisciplinary resources offered in the HRCs contribute to the IMW’s personal development, make them feel more empowered and facilitate their integration into the host country. Understanding IMW’s experiences of the resilience process during the different phases of the migration process could contribute to the development of multidisciplinary and cultural intervention protocols that improve the comprehensive approach to caring for this group in HRCs. In fact, our findings have several implications for improving the programs developed in HRCs. Firstly, promoting resilience can reduce the stigma of IMW and facilitate their integration into the host society. It is important that HRCs promote the confidence of IMW and work on programs that enhance their personal strengths such as self-regulation, conflict resolution, motivation, persistence and optimism. To develop these competences, IMW need support through effective coordination between social, legal and health services. Secondly, in order to help the IMW to change their perceptions, it is crucial to focus on promoting resilience rather than highlighting their trauma. In this regard, we should advocate for policies and services that offer specialized psychosocial support for forced migrants, providing them with more opportunities and greater autonomy.

### 5.1 Implications for practice

Resilience has been shown to be key in helping IMW cope with the migration process in a positive way. Information campaigns in their countries of origin could contribute to redirecting the IMW’s goals and increasing their resilience in the face of such a difficult process. The time the IMW spend in the HRC is perceived as a necessary transition phase to foster resilience, allow them to heal from trauma and prepare them for integration into the host society. Countries receiving IMW should promote financial, material and human resources for HRCs; the European Union should refocus its policies to improve the provisions of these centers. Improving the HRC’s resources and extending the length of time that IMW spend there could contribute to building on their strengths. This study highlights the importance of gender-sensitive approaches to migration policy; IMW exhibit unique vulnerabilities that require tailored support systems to address their specific needs. Building resilience involves transforming the IMW’s painful experiences into a process of learning, adapting their goals and trusting in their abilities. The IMW’s resilience is further boosted by maintaining their faith and values, as well as receiving social support.

## Data Availability

The original contributions presented in the study are included in the article/supplementary material, further inquiries can be directed to the corresponding author.
